# A qualitative evaluation of a nurse-led pre-operative stoma education program for bladder cancer patients

**DOI:** 10.1007/s00520-021-06093-0

**Published:** 2021-03-04

**Authors:** Elizabeth Marie Wulff-Burchfield, Maryellen Potts, Katherine Glavin, Moben Mirza

**Affiliations:** 1 Department of Medicine, University of Kansas Medical Center, Kansas City, KS USA; 2 University of Kansas School of Nursing, Kansas City, KS USA; 3 Department of Urology, University of Kansas Medical Center, Kansas City, KS USA

**Keywords:** Stoma, Urostomy, Patient education, Bladder cancer

## Abstract

**Introduction:**

Radical cystectomy remains the standard of care for muscle-invasive bladder cancer and high-risk non-muscle-invasive bladder cancer. Postoperative ostomy education is common, but patients struggle to maintain self-management practices. A preoperative ostomy education program was developed to meet this need, and we conducted a qualitative study with participating patient-caregiver dyads to evaluate the educational and psychosocial impacts of the program and examine alignment with program objectives.

**Materials and methods:**

A qualitative descriptive study was conducted utilizing a thematic analysis approach. Sixteen patients, eighteen caregivers, and three program educators completed semi-structured interviews from 3 to 18 months post the program. Interviews were audio-recorded and transcribed. Thirteen end-of-course surveys from the initial educational program cohort were transcribed, coded, analyzed; this data was triangulated with patient, caregiver, and educator interviews.

**Results:**

Analysis uncovered three themes: (1) Patient and caregiver motivation to attend the program, (2) attitudes toward this life-changing event, and (3) education. For theme 1, patients and caregivers cited lack of knowledge, fear, and concern about ostomy surgery and care as motivation. For theme 2, there were a variety of attitudes toward the ostomy, ranging from avoidance to acceptance, and a similar breadth of attitudes toward caregiving, with some patients and caregivers describing ongoing dependence and other patients seeking complete independence. For theme 3, the interactive curriculum was determined to be effective, and the patient advocate was cited as the most memorable program component.

**Conclusions:**

A formal preoperative ostomy education program employing an interactive educational approach and featuring a patient advocate can prepare bladder cancer patients and caregivers for ostomy self-management and post-ostomy life.

## Introduction

The standard treatment for muscle-invasive bladder cancer and high-risk non-muscle-invasive bladder cancer is radical cystectomy (RC) with lymphadenectomy and urinary diversion [1]. Patients who undergo RC and ileal conduit must learn to manage an ostomy that requires daily care and manual skills, and they also must cope with the psychosocial impacts that accompany urostomy placement [2]. Many patients are not adequately prepared to cope with the changes of post-ostomy life, which can lead to extra clinic visits, stoma-related complications (e.g., rashes, stomal stenosis), and reported feelings of being unequipped to deal with new changes to their body. These difficulties have often led to a decrease in patients’ post-operative health related quality of life [2].

Challenges of living with a stoma include learning how to perform daily care, manage incontinence and sexual dysfunction, how to cope with issues around body image, self-esteem, and maintaining regular daily activities [3, 4]. Problems with diet and clothing are common, and these can have professional consequences [5]. Upon seeing their stoma for the first time, patients often react with disgust, shock, or feeling disconnected from their own body [5]. Such feelings are barriers to patients attaining competence and confidence with ostomy self-care, which is essential for quality of life (QOL) [6] and adaption to post-stoma life [2, 7]. Providing information and guidance about physiologic or functional changes arising from medical procedures facilitates patients feeling a sense of control regarding their health conditions and bodily changes [8].

Research conducted about stoma education programs generally illustrates a positive trend in psychological outcomes such as QOL, self-care, and self-efficacy [9–11]. Ostomy educational program curricula focus on recognized patient needs, such as handling the stoma, managing appliances, diet, and sexuality issues; programs utilize small group sessions and lay and peer teachers[10]. However, while postoperative, hospital-based instruction regarding urostomy self-management is widespread and remains the mainstay of urostomy education [3, 4], patients do not tend to retain education delivered post-operatively as much as would be desired [12, 13] and struggle to maintain self-management practices [14]. Caregiver support cannot reliably overcome the challenges that arise when stoma education is confined to the postoperative timeframe because caregivers are often focused on the immediate physical needs of the patient rather than on reinforcing stoma instruction. Therefore, educational strategies must address patients’ psychosocial concerns, including preoperative timing [15]. Preoperative stoma education has been shown to significantly improve self-efficacy [16] and reduce patients’ postoperative anxiety; thus, this indirectly supports ostomy self-care by removing anxiety, which is itself a barrier to learning [17].

For years, all of these issues have been part of the rhythms of daily life for centers with a high volume of radical cystectomy cases, with post-cystectomy patients and their caregivers calling and requiring ostomy care visits frequently regarding skin care, ostomy supplies, ostomy adjustment, and other topics covered in postoperative education. Due to these clinical needs and urologists’ and ostomy nurses’ understanding of patients’ and caregivers’ poor retention of postoperative ostomy education and the importance of patient and caregiver competence and self-efficacy regarding ostomy care, a prospective pilot study was undertaken at a single academic medical center to assess the effects of a preoperative urostomy educational intervention, “Stoma Bootcamp” (SBC). This parent study evaluated patients’ ostomy adjustment and self-efficacy; the quantitative results of this parent study were reported elsewhere. In adjunct to this parent study, we conducted a qualitative evaluation study to characterize the value of the SBC to patients and caregivers. Our qualitative study examined patients’ and caregivers’ perceptions of the psychosocial and educational effects of the program and evaluated concordance with the intentions of the SBC educators who developed content and delivery methods.

## The Stoma Bootcamp intervention

The preoperatively delivered Stoma Bootcamp (SBC) was developed by a team of specialists including a urology nurse practitioner, an ostomy nurse, and a project coordinator at the University of Kansas Health System (KUHS) Urology Department to provide participants with the knowledge to effectively adapt to life after surgery. The Stoma Bootcamp included components suggested by previous research, such as psychosocial care, pre-operative timing, and lay and peer teachers. Our urologic oncology and ostomy teams perceived that patients and caregivers needed more preparation for the procedure and post-stoma life to help alleviate patient and caregiver anxiety and fear.

The stated goal of the SBC was to improve patients’ and caregivers’ abilities to care for the stoma physically and psychologically. Content of the program included such topics as the purpose of an ostomy, the procedure to create an ostomy, the postoperative experience and postoperative care, ostomy appliances, daily living, and a plethora of resources for help with ostomy support, from physician phone numbers to ostomy support groups and ostomy product manufacturers. The SBC was held in person beginning in 2018 at the KU Cancer Center; persons undergoing urostomy due to bladder cancer and their caregivers were invited to attend a 2-h SBC prior to their procedure on the recommendation of their healthcare team. The format of the program included lecture, audiovisual presentations, hands-on activities with pouching supplies, use of an anatomical model to illustrate the surgical procedure, use of a soft stoma doll for take home use with younger family members, the presence of a patient advocate – a previous SBC attendee who served as a model of post-ostomy life, answered participant questions, and demonstrated what a stoma looked like on a real person. A stoma product manufacturer’s representative was also present to answer questions. Programming was delivered by the urology nurse practitioner, the ostomy nurse, and the project coordinator. Take home materials included an illustrated booklet of the content, sample pouching supplies from a manufacturer’s representative, the stoma doll, and a DVD of stoma-related information. The SBC is offered twice per month, and since March of 2020, a virtual option has been added. Average participation is 5 patients per month in the virtual format and 3 patients plus 3 caregivers per month for in-person option.

KUHS Urology Department investigators conducted a randomized pilot study to assess the feasibility of the SBC and to generate preliminary data regarding the effect of the SBC on ostomy adjustment utilizing the patient-reported outcome measure, the Ostomy Adjustment Scale, and other quantitative perioperative outcomes. The goal of the present study was to qualitatively explore the effect of the Stoma Bootcamp on patients and caregivers to help illustrate impacts not captured by quantitative data and to refine the curriculum in order to better meet patients’ and caregivers’ needs for urostomy preparedness and coping.

## Materials and methods

### Design

This study utilized a descriptive qualitative methodology with a thematic analysis approach [18]. Thematic analysis is a method that permits investigators to identify, analyze, and report patterns found in qualitative data [18]. Three sets of individual interviews were conducted: bladder cancer patients who attended the SBC (*n* = 16), patients’ identified caregiver who attended the SBC (*n* = 18), and educators who devised and delivered the content of the SBC (*n* = 3). Participants were interviewed from 3 to 18 months. The study followed the Consolidated Criteria for Reporting Qualitative Research (COREQ) guidelines, a 32-item checklist for interviews and focus groups [19]. This research was approved by the Institutional Review Board of the University of Kansas Cancer Center (#145269).

### Participants

Patient, caregiver, and educator consent and interviews were conducted by MP with the assistance of EWB. MP had no prior relationship with study participants. EWB had previously participated in the medical care of two participants.

The Stoma Bootcamp was held beginning in 2018 at The University of Kansas Cancer Center, a National Cancer Institute (NCI)-designated cancer center (P30 CA168524). For this study, a purposeful sampling strategy was utilized to select participants whose information would align with the study’s purpose [20], i.e., offer evaluative information about the Stoma Bootcamp intervention. Patients and caregivers were recruited from SBC participants. Inclusion criteria were age ≥ 18 years, ability to speak English, and attendance at one SBC session prior to radical cystectomy for high-risk, non-muscle-invasive or muscle-invasive bladder cancer. Persons with metastatic cancer at the time of cystectomy were excluded and all patients were clinically node negative. Patient participant characteristics are shown in Table 1. The SBC coordinator identified eligible patients and mailed letters informing them of the study. Researcher MP contacted, consented, and interviewed patients and caregivers by phone to avoid any potential bias with the patients’ oncologist, EWB. Patients who participated in the interviews identified a caregiver who attended the SBC with them—most often a spouse or family member—and referred this caregiver to the research team. Two patients who were approached for an interview declined participation. Approximately 20% of patients who received phone messages from the research team were ultimately not reached. Three educators (SBC coordinator, ostomy nurse, and urology nurse practitioner) who delivered the program participated; they were contacted by EWB, and video interviews were conducted via the secure UKHS link. The SBC patient advocate declined participation. No compensation was provided to any participant.

**Table 1:** Demographics for patient participants

Age	Years (range)
Mean age	71 (52–89)
Gender	*N* (%)
Female	7 (24%)
Male	22 (76%)
Marital status	*N* (%)
Single	1(3.4%)
Married	24(82.8%)
Divorced	2 (6.9%)
Widowed	2 (6.9%)
Disease status	*N* (%)
Non-muscle-invasive bladder cancer	8 (27.6%)
Muscle-invasive bladder cancer	21(72.4%)
Clinical Stage** (pre-SBC)	*N* (%)
Tis	2 (6.9%)
Ta	3 (10.35%)
T1	3(10.35%)
T2	19 (65.5%)
T3	2 (6.9%)
Performance status (ECOG*)	*N* (%)
0	19 (65.5%)
1	7 (24.15%)
2	3(10.35%)
Neoadjuvant chemotherapy	*N* (%)
None	14 (48.3%)
Cisplatin + Etoposide	1 (3.4%)
Cisplatin + Gemcitabine	7 (24.15%)
Dose-dense MVAC***	7 (24.15%)

*Eastern Cooperative Oncology Group (ECOG) performance score

**American Joint Committee on Cancer (AJCC), 8^th^ edition staging

***Methotrexate, Vinblastine, Doxorubicin, Cisplatin

### Data Collection

Semi-structured interview guides were developed using the theoretical framework of Engel’s Biopsychosocial Model (BPS) [21]. One guide was geared towards patients and a parallel guide towards caregivers regarding motivation to attend the SBC and its effect. A similar guide was developed for the SBC educators regarding the motivation to create the SBC program, content and delivery decisions, and perceptions regarding participant engagement. Table 2 provides questions from the interview guides. All interviews were recorded and individually conducted with patients, caregivers, and educators until thematic saturation was reached. The majority of patient and caregiver interviews lasted 15 min or less; educator interviews lasted approximately half an hour apiece. Recordings were transcribed verbatim and de-identified. An additional data source was a set of written surveys delivered to 13 participants at the beginning of the original parent study. Surveys were designed in a manner consistent with other patient satisfaction and quality improvement metrics employed at the study institution related to clinical care. This survey contained 5 yes/no questions and 4 open-ended questions and was delivered to patients and caregivers at the first clinic visit following the SBC and radical cystectomy admission. Survey participants were not included in the interviews. Transcripts and de-identified surveys were uploaded to a secure UKHS server.

**Table 2:** Selected interview questions and survey items

Questions			
Patient	Caregiver	Educator	Survey
What kind of concerns did you have before going to the SBC?	What kind of concerns did you have when you learned [person with ostomy] needed ostomy surgery?	From your perspective, what need did you think the SBC would fill?	Did you have any ostomy knowledge prior to your participation in the study? If so, can you give us more information regarding this?
Did you have any concerns about: The surgery itself? What would happen after surgery? About living with an ostomy?	Did you have any concerns about: The surgery itself? What would happen after surgery? About living and caring from someone with an ostomy?	How did you determine the content of what you offered at the SBC? What methods did you use to deliver the content?	What challenges or problems did you face post-operatively regarding your ostomy and recovery?
Did the SB help you with your concerns? How?	Did the SBC help you with your concerns? How?	From your observations, how do you think the SBC affected participants?	Did you find the information you were provided during the boot camp helpful prior to your surgery? How well did you understand your surgery at the end of the boot camp?
In your opinion, what was most helpful about the SBC?	In your opinion, what was most helpful about the SBC?	In your opinion, what was the most compelling or important part of SBC for the participants?	Did you enjoy attending the boot camp? What parts did you enjoy the most? What parts did you enjoy the least?
Was there anything missing from the SBC, in your opinion? Any suggestions for improvement?	Was there anything missing from the SBC, in your opinion? Any suggestions for improvement?	Have you made any changes in the Boot Camp since it began? What sorts of changes?	Did you find the information provided to you on the class handout to be helpful? Any parts of that you thought needed improving?

### Data Analysis

Patient and caregiver transcripts were individually coded by MP and EWB by hand to develop the code tree. Coding utilized a deductive/inductive approach consisting of deductive coding based on concepts connected to the interview questions, and an inductive coding that allowed new information to emerge [11]. A constant comparative coding method was used by both investigators who began with open coding to generate initial descriptive codes, compared these codes to earlier codes, revised and recoded, and then moved to more focused, analytical coding following the same iterative process [22]. The constant comparative method also meant that one investigator reviewed transcripts coded by the other team member, discussed the evolving codes, and then refined and developed emerging codes until between a 85–90% consensus about the code tree was reached by both team members.[23] When the qualitative software Dedoose became available to the investigators, educator transcripts and patient surveys were imported into Dedoose for analysis and coded by MP. A separate but parallel code tree was developed for educator and survey transcripts due to transcript content. The process of thematic analysis allowed investigators to organize similar codes into categories, the first step towards identifying patterns in the data and explanations for these patterns [23]. Themes and sub-themes emerged from the categories as meaning was attached to the categories across the four main data sources and exemplar quotations were identified for each theme.

## Results

Sixteen patients, eighteen caregivers, and three SBC educators participated in this study. Thirteen written surveys from participants collected during the original study were also utilized in the data analysis. Table 3 illustrates codes, categories, themes, and exemplar quotations.

**Table 3:** Codes, categories, and themes

Exemplar quotations	Code	Category	Theme
Theme 1			
*Patient:* “I had no information about those stomas before the class. I didn’t even really know what it was.” (Survey participant H) *Educator:* “[patients] look at [the stoma] and they think, this is my intestine sticking out of my belly... for that day, we cannot do any education. It’s too shocking [and] they can’t really look past that.” (Ostomy nurse)	Need for knowledge as motivation	Motivation	Motivation to attend Stoma Bootcamp—stepping into the unknown
*Caregiver:* “…we were thrown into bladder cancer and stoma so quickly that we were just desperate for information. We were scared …and so [SBC] was wonderful.” (Caregiver 102) *Educator:* “We thought the class would really help reduce that anxiety and stress” (Bootcamp coordinator)	Fear as motivation		
*Patient:* “I had more concerns about life after boot camp… But I was told that I could go fishing for about two years **or** [with] surgery, I could have maybe 20 years. So, that convinced me upfront ...” (Patient 15)	Concerns as motivation		
Theme 2			
*Patient:* “I hadn’t wanted to face [the ostomy] up until then. So, it had to happen at some point before the surgery actually took place, so [the SBC] was useful.” (Patient 12) *Educator:* “The folks that have come to this class, that shock part is gone. They've seen [a stoma in class]. Obviously haven't seen their own yet, but …they talked about it with their spouse, they have these [kits, tools] … And then it’s not as scary coming back.” (Ostomy nurse)	Attitude towards the stoma/ostomy bag	Attitude	Attitudes toward this life changing event—adjusting one’s worldview
*Patient:* “Well I can’t change the bag by myself, I have to have my son or my daughter or my partner change it for me. Even if I’m standing in front of the mirror – it’s not like I haven’t tried.” (Patient 20) *Patient:* “And I’ve done it all myself since day one. My wife wanted to help at the very beginning, but she’s too meticulous.” (Patient 8) Caregiver: “I felt sorry for [my husband]… I had offered to help him try to put it on, but he says no, not to. And at the class, maybe they should say that if you have problems, maybe your caretaker could assist you in putting it on.” (Caregiver 121) *Educator:* “…patients themselves would always ask questions like, ‘I don’t want to burden my loved one.’ “(Bootcamp coordinator) *Educator*: “We …stress independence ... And that you can do this yourself.” (Ostomy nurse)	Adjusting towards caregiving (of self or for caregiver)		
*Patient:* “They also marked my place where my stoma [would be during class] and on the day I showed up for surgery, they came in to mark my stoma, I said, but I’ve already had it. And so, …one less thing they have to deal with that day.” (Patient 2) Educator: “[Undergoing ostomy surgery] can be really anxiety-provoking, so this class [was about] quality of life, [alleviating] anxiety, and just really having that good, fundamental knowledge about the surgery and the supplies you’ll need for the rest of your life.” (Bootcamp coordinator)	Preparedness in stoma care		
Theme 3			
*Patient:* “Just seeing there’s lots of people besides me there that have the same problem as I have. Feeling not alone when you’re faced with something like that …” (Patient 13) *Caregiver:* “The doctor can explain things to you, but then actually seeing the actual urostomy bags and how it works, and how they put them together …that was real helpful.” (Caregiver 113) *Educators:* “We thought it was important that [participants] would get some hands-on, so they would see what a pouch was, what they would need to change a pouch.” (Urology advance practice provider [APP])	Teaching delivery style	Education	Education—providing the tools needed to live this new life
*Patient:* “Most helpful was just watching somebody [patient volunteer with stoma] who had already gone through it and seeing them doing well.” (Patient 14) *Caregiver:* “…they also gave us a little doll that we could take home to all the grandkids. That was really helpful with explaining, like, Grandma’s going to have this on her now.” (Caregiver 102) *Educators:* [Patient volunteer] showed them that it’s a trial and error with some things… Certain things like that that we could not provide because we haven’t lived through it, she really provided that aspect to patients.” (Bootcamp coordinator)	Resources/personnel		
*Patient:* “I was scared to death, and when it was all said and done, it just seemed like it would be an easy thing. [The SBC] was very informative.” (Patient 20) *Caregiver:* “I think the stoma boot camp helped us… It gave us some idea of what to expect. … After we got home, a lot of [the information] was spot on... so that helped us out a lot.” (Caregiver 117) *Educators:* “I think that there were some people that were overwhelmed, and …we would …talk to them. ‘Hey, are you doing okay? Did you have any other questions? If you have questions, [call this] number.’” (Urology APP)	Program expectations		

### Theme 1: Patient and caregiver motivation to attend the Stoma Bootcamp—stepping into the unknown

Categories leading to this first theme included the need for knowledge, fear, anxiety, and concerns about living with an ostomy. Motivation to attend the SBC was a need for information. One educator stated: “There was a significant knowledge deficit that the patients and the families were experiencing prior to coming in for the radical cystectomy” (Urology Advance Practice Provider [APP]). A patient stated: “the more information you have about [the stoma], the better equipped you are to handle it” (Patient 2). Survey respondents agreed about their lack of knowledge; educators indicated that lack of knowledge led patients to react with shock upon seeing the stoma for the first time which then obviated any potential for education on how to care for it. The shock of diagnosis and accompanying fear and anxiety about surgery also spurred patients and families to attend the SBC.

Patients and caregivers were concerned about caring for the stoma and about QOL after ostomy surgery. A caregiver said: “I had concerns just about [my mom’s] lifestyle change, if it was going to change her lifestyle and keep her the mom that we had before the cancer or if it was going to be [worse] after” (Caregiver 102). Educators believed that providing “fundamental knowledge about the surgery and the supplies you’ll need for the rest of your life” would quell fear and improve expectations about life quality (Bootcamp coordinator).

### Theme 2: Attitudes toward this life-changing event—adjusting one’s worldview

Categories leading to this second theme were attitudes towards the stoma and the “new self,” and attitudes towards caregiving. Attitudes regarding the first category fell over a broad spectrum. Some participants described acceptance of the stoma as a necessary or inevitable step toward pursuing aggressive cancer care. One caregiver stated, “ [the SBC] was just part of the process to get to the end, which was to get him the ostomy so he could get rid of the bladder that had cancer” (Caregiver 108). Survey feedback aligned with this attitude. For others, the SBC served as a wake-up call to confront the realities of their impending life change which they had previously avoided.

Participants exhibited a wide range of attitudes toward caregiving. Some noted an undercurrent of dependence between patient and caregiver as stated by one patient: “My greater concern was how to care for [the stoma] and how to take care of it after surgery was over. And even now, my husband still helps me” (Patient 17). Some patients and caregivers described a strong desire of patients to achieve independence in stoma care. Educators emphasized self-sufficiency with stoma care: “…we have a lot of husbands that say, ‘Oh, my wife’s just going to do it.’ And so…we push independence” (Ostomy nurse). Patients and caregivers also learned when it was appropriate to offer help and support, as well as when it was not necessarily wanted.

Participants generally agreed that preparedness was important. However, participants with different roles described different aspects of preparedness. For example, patients reported feeling equipped to manage the technical aspects of post-ostomy life and caregivers noted a more global sense of preparedness for the operation and life changes to come: “But I know when we came home [from the SBC], ready for the surgery and not so concerned about the bag that she’s going to have to wear” (Caregiver 119). Educators designed the course to cultivate a sense of preparedness regarding technical abilities as well as impending life changes.

### Theme 3: Education—providing the tools needed to live this new life

Three categories informed the third theme: teaching delivery style, resources and personnel, and program expectations. Educators wanted the SBC to be interactive for patients and caregivers and chose delivery methods that ensured hands-on practice with stoma-related materials. A caregiver stated: “The doctor can explain things to you, but then actually seeing the actual urostomy bags and how it works, and how they put them together and all that, I thought that was real helpful” (Caregiver 113). The teaching was intentionally in-person and several patients commented on the group process: “Just seeing there’s lots of people besides me there that have the same problem as I have. Feeling not alone when you’re faced with something like that…” (Patient 13).

The educators delivered practical information, provided curated resources, and offered participants the chance to see a stoma on a living person. Educators utilized stoma pouching kits donated by industry representatives and soft dolls with stomas for patients to take home, as well as anatomical models that illustrated the surgical procedure and urinary reconstruction. A caregiver stated: “The doctor and some others kind of told us what to expect and what was going to take place, but it was neat to see it up close...” (Caregiver 117). Analysis revealed that the most effective, memorable, and reassuring aspect of SBC was the patient advocate—a previous attendee of the SBC with who volunteered to share her experience of a living with a stoma. Educators said that the patient advocate “show[ed] people that there is life after getting a stoma” (Urology APP). A patient summed up by saying: “Most helpful was just watching somebody who had already gone through it and seeing them doing well” (Patient 14).

Program expectations were met from the perspective of patients and caregivers as well as educators who perceived that participants received information positively. Praise for the educators was universal.

## Discussion

The findings of our Stoma Bootcamp evaluation illustrated that bladder cancer patients and caregivers felt that the cancer diagnosis and the prospect of radical cystectomy were unknown and unexpected variables in their lives, that in general, their expectations of post-ostomy life moved from anxiety to confidence as a result of the SBC program, and that the education they received was effective and timely. These results echo those of Jensen, et al, which found a statistically significant improvement in scoring on the Urostomy Education Scale when assessing ostomy “efficacy” at 35 days and 120 days postoperatively [16]. Our SBC program, unlike that of Jensen, et al, included a patient advocate with a stoma, and we included psychosocial and program evaluation findings.

Our data analysis yielded that patients’ individual coping styles seemed to play a role in their level of acceptance of the upcoming life change; some patients were motivated to master stoma care and others recognized the importance of partnering with their caregiver, usually a spouse. Overall, patients were willing to care for their stoma and were intrinsically motivated to seek independence. This attitude differed from the perceptions held by the educators, indicating that patient motivation and attitude may be more nuanced than what is apparent from brief interactions with healthcare professionals, even those with ostomy expertise. Caregivers wanted to help with stoma care when needed, but not all patients were willing to accept this. Therefore, including a communication module in the SBC could help support the patient-caregiver dyad.

The program was well received by patients and caregivers, which aligned with the educators’ perceptions of the program’s success. SBC participants lauded the knowledge and expertise of the educators and had few criticisms. The highlight of the program for patients and caregivers was learning from the patient advocate who illustrated the possibilities of living with a stoma. Participants were empowered by the practical, technical knowledge they received, as well as by opportunities to practice with ostomy bags. Another cited value was the presence of a ostomy supply company representative. This finding of enhanced education that included practical knowledge and hands-on practice, and the value of preoperative experience which included a tour of the facility, aligned with the program recommendations presented by McMullen et al. This study employed a “user-centered design” which focused on the preferences of the “users” to develop patient- and caregiver-centered interventions for patients undergoing complex surgery such as urostomy [24]. The consensus among participants in the SBC was that the educators were successful in delivering information in a manner that addressed the learning preferences of most participants. The SBC employed a variety of approaches to deliver information, from small group discussion to online sources. The least-preferred content delivery style was packets of printed information.

Since implementing SBC, educators have recognized improvement in patient preparedness, which provides face validity for the methods of delivery and course content. Krouse, et al, also employed a varied approach to teaching (interaction, discussion, demonstration) in a pilot trial of a post-operative multi (5) session ostomy self-management educational program for people with colostomies and urostomies [25]. Findings included sustained improvement in patient activation as well as health-related quality of life (HRQOL) and depressive symptoms; however, participants indicated that they wanted more hands-on training [25]. Our Stoma Bootcamp educational program was different from other stoma education interventions in that it was delivered preoperatively to overcome the problem of patient and caregiver knowledge retention due to the shock value of ostomy surgery. It also incorporated hands-on learning and skills practice, in addition to having the presence of a patient advocate who shared “real life” problems and solutions of living with a stoma. These intervention components may be a useful model for other institutions to consider as they develop stoma education programs.

### Limitations

Demographics were ethnically homogeneous, attributable to patient population in the study institution’s catchment area. Most patient participants identified with their birth sex and most partners were heterosexual. The Stoma Bootcamp program may need modifications in order to meet the needs of the broader US population. This program was developed and implemented prior to the SARS-CoV-2 pandemic, and therefore further study is needed in order to successfully modify the content and delivery style to accommodate social distancing and other measures for this vulnerable patient population.

## Conclusions

Radical cystectomy with ileal conduit reconstruction for bladder cancer is a life-altering experience, and both patients and caregivers experience fear and anxiety about the operation and post-stoma life and care. A formal stoma education program like Stoma Bootcamp can prepare patients and caregivers for the impacts of a urostomy, and preoperative timing is essential. Our data demonstrated that these preoperative educational interventions need to be interactive and hands-on whenever possible and that a patient advocate is an essential component of the educational curriculum and team. Future work includes evaluating patients’ and caregivers’ health-related quality of life (HRQOL) prior to attending the SBC and the urostomy procedure and after to longitudinally assess the effect of the educational program on HRQOL. Utilizing validated HQROL questionnaires such as the Bladder Cancer Index and the Functional Assessment of Cancer Therapy - Vanderbilt Cystectomy Index to help assess the impact of a pre-operative educational program could help refine such instruments further regarding their use with patients undergoing urostomies.

## Data Availability

We have full control of all primary data and agree to allow the journal to review these data if requested

## Code availability

N/a
